# Welding of High Entropy Alloys—A Review

**DOI:** 10.3390/e21040431

**Published:** 2019-04-24

**Authors:** Jing Guo, Cong Tang, Glynn Rothwell, Lisa Li, Yun-Che Wang, Qingxiang Yang, Xuejun Ren

**Affiliations:** 1 Department of Mechanical and Maritime Engineering, Faculty of Engineering and Technology, Liverpool John Moores University, Liverpool L3 5UG, UK; 2 Department of Civil Engineering, National Cheng Kung University, Tainan 701, Taiwan; 3 State Key Laboratory of Metastable Materials Science and Technology, College of Materials Science and Engineering, Yanshan University, Qinhuangdao 066004, China

**Keywords:** high-entropy alloys, welding, Hall–Petch (H–P) effect, lattice constants

## Abstract

High-entropy alloy (HEA) offers great flexibility in materials design with 3–5 principal elements and a range of unique advantages such as good microstructure stability, mechanical strength over a broad range of temperatures and corrosion resistance, etc. Welding of high entropy alloy, as a key joining method, is an important emerging area with significant potential impact to future application-oriented research and technological developments in HEAs. The selection of feasible welding processes with optimized parameters is essential to enhance the applications of HEAs. However, the structure of the welded joints varies with material systems, welding methods and parameters. A systemic understanding of the structures and properties of the weldment is directly relevant to the application of HEAs as well as managing the effect of welding on situations such as corrosion that are known to be a service life limiting factor of welded structures in conditions such as marine environments. In this paper, key recent work on welding of HEAs is reviewed in detail focusing on the research of main HEA systems when applying different welding techniques. The experimental details including sample preparation, sample size (thickness) and welding conditions reflecting energy input are summarized and key issues are highlighted. The microstructures and properties of different welding zones, in particular the fusion zone (FZ) and the heat affected zones (HAZ), formed with different welding methods are compared and presented in details and the structure-property relationships are discussed. The work shows that the weldability of HEAs varies with the HEA composition groups and the welding method employed. Arc and laser welding of AlCoCrFeNi HEAs results in lower hardness in the FZ and HAZ and reduced overall strength. Friction stir welding results in higher hardness in the FZ and achieves comparable/higher strength of the welded joints in tensile tests. The welded HEAs are capable of maintaining a reasonable proportion of the ductility. The key structure changes including element distribution, the volume fraction of face centered cubic (FCC) and body centered cubic (BCC) phase as well as reported changes in the lattice constants are summarized and analyzed. Detailed mechanisms governing the mechanical properties including the grain size-property/hardness relationship in the form of Hall–Petch (H–P) effect for both bulk and welded structure of HEAs are compared. Finally, future challenges and main areas to research are highlighted.

## 1. Introduction

High-entropy alloys (HEAs) are based on the promising alloy design ideas of configurational entropy maximization, which offer great flexibility in developing different material systems [1,2,3,4,5,6]. Compared with the classic alloys, HEAs possess better performance in areas such as hardness, wear resistance, fatigue and resistance to corrosion and oxidation [3,4,5,6] as well as novel physical properties [2,3]. In general, HEA has high intrinsic strength and ductility, and the low diffusion rate of HEAs at high and low temperatures makes their applications in harsh condition possible [1,7,8]. There are many different material systems and processing methods developed in the past decades, which offer great flexibility in materials and processing methods selection for different conditions with a suitable welding process. In the meantime, the complexity of the material systems requires development of detailed understanding of the material behavior in different manufacturing processes including both hot and cold working operations. Joining of HEAs in similar or dissimilar material systems is increasingly important for expanding the applications of HEAs [9], among which welding process of HEAs is challenging due to the complex chemical, physical, and mechanical nature of welding and the need for tailoring the properties and structures of the weldment for complex loading conditions in similar and dissimilar welding.

**Figure 1:**
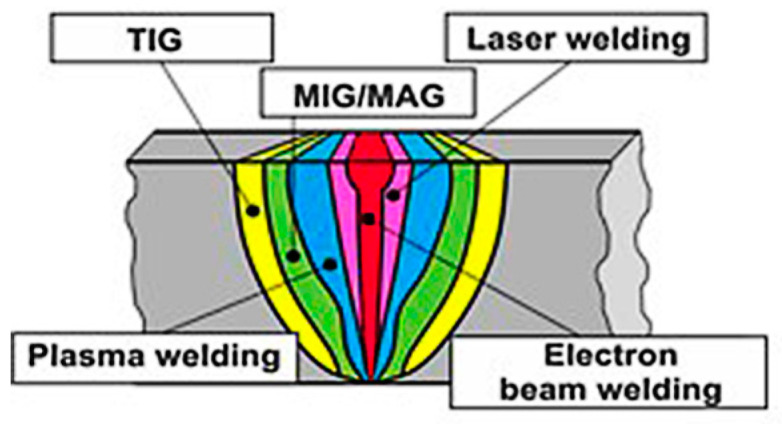
Welded zones formed by different welding methods. [http://is.gliwice.pl/en/strona-cms/electron-beam-welding-laboratory].

HEA can be produced by many different routes such as liquid processing approaches (e.g., arc melting, Bridgman solidification, atomization and laser cladding), additive manufacturing, mechanical alloying (e.g., powder metallurgy), mixing elements of the vapor state including sputter deposition, atomic layer deposition and vapor phase deposition [1,10,11,12,13,14,15,16,17,18]. Welding is a complex process due to the fact that many conditions or factors affect the welding process. The quality of the welded joints can be assessed by the microstructure of the welding zones formed and mechanical structural integrity of the weldment as well as corrosion and fatigue [12]. There are many different welding processes such as arc welding, laser welding, friction stir welding, electron beam welding, electrical resistance spot welding as well as other processes such as electroslag welding, vacuum diffusion welding, etc. [9,12,13,14,15,16]. These welding processes differ significantly in the welding mechanism, working principles of the equipment and efficiency, but each has been found to have wide applications in different industries and application conditions. The energy input and the maximum temperature could be many orders of magnitude different from each of the welding methods, which directly influence the elemental and material behavior such as evaporation, melting and cooling. For example, the heat density ranges from 10^5^–10^6^ W/cm^2^ for gas metal arc welding or plasma arc welding to 10^7^–10^8^ W/cm^2^ for laser or electron beam welding [15,16]. The structural zones formed (Figure 1) after different welding processes could be significantly different in geometry, size, microstructure and compositions, which is dependent on many factors such as the behavior of material systems (elements, evaporation, melting, flow, phase changes, cooling, etc.), the original structures of the workpiece, size of the specimen and the welding parameters, etc. The structure and properties of the welded joints (such as size/shape of the welding zones, the hardness distribution, residual stress, defects, etc.) also affects the performance of the overall joints under different modes of loading and service conditions [17]. These directly influence the suitability of each welding process on joining different HEAs, the application of HEAs and future developments in related areas [1,5,6,18].

In this paper key recent work on welding of HEAs is systematically reviewed. The main works on welding of different HEAs with different welding techniques are presented. The experimental details including samples preparation, sample size (thickness) and welding conditions reflecting the speeds and energy input are summarized. The different welding zones formed with different welding methods and the microstructures are compared. The hardness change of the fusion zone (FZ) and heat affected zones (HAZ) in different welding processes is compared and linked to the material systems. The mechanism of microstructure and property changes in the FZ and HAZ and their influence on the mechanical properties is discussed. Data on the Hall–Petch (H–P) effect (grain size-property/hardness relationship) for both bulk and welded structure of HEAs is compared. The future challenges and main areas to research are highlighted on the weldability of HEAs, welding process selection and potential effects of the microstructure and properties in different welding zones of HEAs on their applications.

## 2. Welding of HEAs in Different Welding Processes

### 2.1. Arc Welding of HEAs

**Figure 2:**
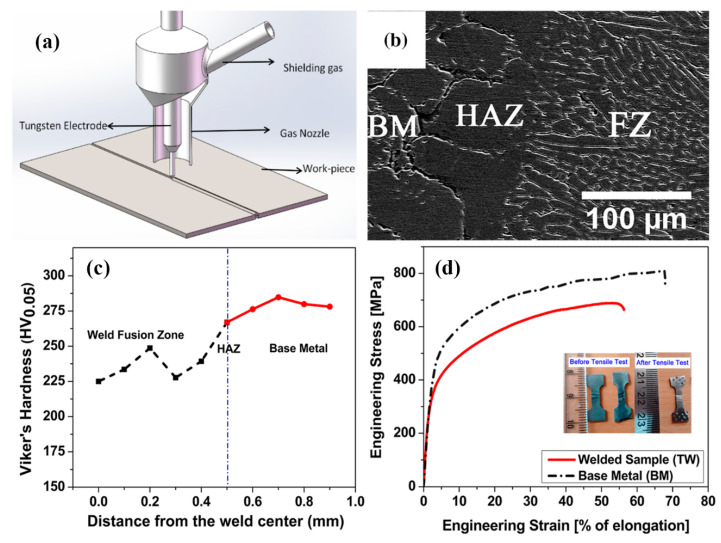
Structure and hardness of the welded zones and engineering stress-stain curves of the welded samples of Al0.5CoCrFeNi high entropy alloy (HEA) [19]: (**a**) Schematic of gas tungsten arc (GTA) welding process, (**b**) Scanning electron micrographs of base metal (BM)–heat affected zone (HAZ)–fusion zone (FZ) interfaces, (**c**) Microhardness profile on the surface of the welded sample and (**d**) Engineering stress–engineering strain curve for the BM and welded sample.

Arc welding is one of the most common welding methods, in which the energy from an arc of electric current between the material and a consumable/non-consumable electrode stick (either) is employed to melt the workpiece(s) with/without a protective atmosphere. Typical arc welding includes shielded metal arc welding (SMAW), gas tungsten arc welding (GTAW) (also known as TIG (Tungsten inert gas)), gas metal arc welding (GMAW), flux-cored arc welding (FCAW), submerged arc welding (SAW) [15,16]. Most of the work on welding HEAs has been focused on gas tungsten arc welding (GTAW) [19,20]. In the welding process, a non-consumable tungsten electrode is used to produce the weld by melting the base metal (Figure 2a), and the weld area is protected from atmospheric contamination by an inert shielding gas such as argon or helium. In the work by Sokkalingam et al [19], two Al0.5CoCrFeNi–HEA plates (homogenized at 1423 K for 24 hours followed by furnace cooling) of a thickness of 2.5 mm were butt-welded with a current of 40 A and voltage of 12 V at a welding speed of 80 mm/min. The FZ and HAZ formed exhibited distinctively different structures from the base metal (BM) (as shown in Figure 2b). The dominant structure in the FZ mainly consisted of fine elongated grains with diameter of 8–12 µm and lengths of 80–120 µm. The HAZ had a distinctive coarse grain structure with grain boundaries delineated by the body centered cubic (BCC) phase. Vickers micro-hardness tests across the three zones revealed an inferior hardness of the FZ and HAZ compared to the BM, particularly at the center of the welding pool (Figure 2c). Tensile tests of welded sample perpendicular to the welding seam showed that both the yield strength (YS) and the ultimate tensile strength (UTS) were reduced after welding, but the welded sample has maintained a high proportion of the ductility (Figure 2d), which is better than samples with other welding processes (detailed in the following sections).

**Figure 3:**
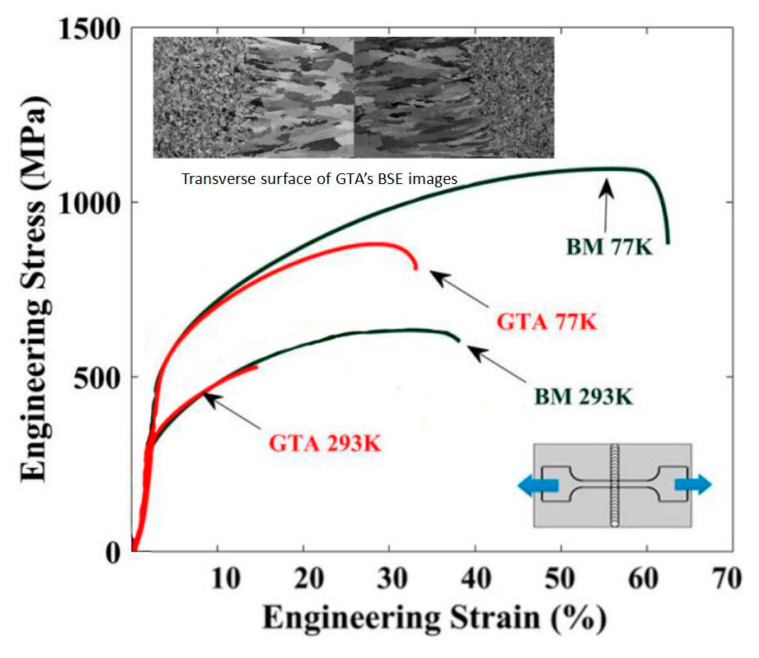
Structure of the welding zones and tensile test results of welded joint of CoCrFeMnNi alloy by GTA [20].

CoCrFeMnNi system is another major HEAs system with significant potential in structural applications [20,21,22,23]. Wu et al [20] investigated the welding of CoCrFeMnNi HEAs with low-energy-density, high-heat-input gas tungsten arc (GTA). The raw material of the workpiece was arc-melted and drop-cast into an ingot of 25.4 mm × 12.7 mm × 127 mm, a rolling operation along the longitudinal direction was then performed in air at room temperature to reach a final thickness of 1.6 mm. The sheets were annealed at 900 °C for 1h to obtain an equiaxed microstructure. Buttwelding of two pieces of the sheet alloys were performed at a power level of 8.4 V and 75 A with a welding speed of 25.4 mm/min. The FZ of the welded joints exhibited a centerline grain structure of large columnar grains grown from the fusion lines to the centerlines (Figure 3). Tensile tests of the specimen at 293 K and 77 K showed that the effective YS of the GTA weld was close the BM, but the UTS was significantly lower, in particular for the low temperature test. The ductility of the GTA sample was maintained over 50% (Figure 3). The difference in the microstructures of the FZ between the two material systems (i.e., Al0.5CoCrFeNi and CoCrFeMnNi) is apparent, both showed columnar grain structure, but the effect of the welding on the mechanical response is different. The FZ of Al0.5CoCrFeNi–HEA (Figure 2d) showed a drop in YS and UTS, and maintained high proportion of the ductility against the BM; while the drop in YS for CoCrFeMnNi (Figure 3) was relatively limited but there was a clear drop of the UTS, but it had only maintained about half of the ductility. The work also revealed limited depletion behavior of Mn in the weld zone, which is different from welding of high-Mn stainless steels [24]. Nano-twins (Σ3) common to CoCrFeMnNi HEA and other face-centered cubic (FCC) metals and alloys with low stacking fault energy were also observed [25]. The nano-twins and twin bundles formed during deformation had significantly beneficial effect on mechanical properties such as tensile strength.

### 2.2. Laser Welding/Laser Beam Welding

**Figure 4:**
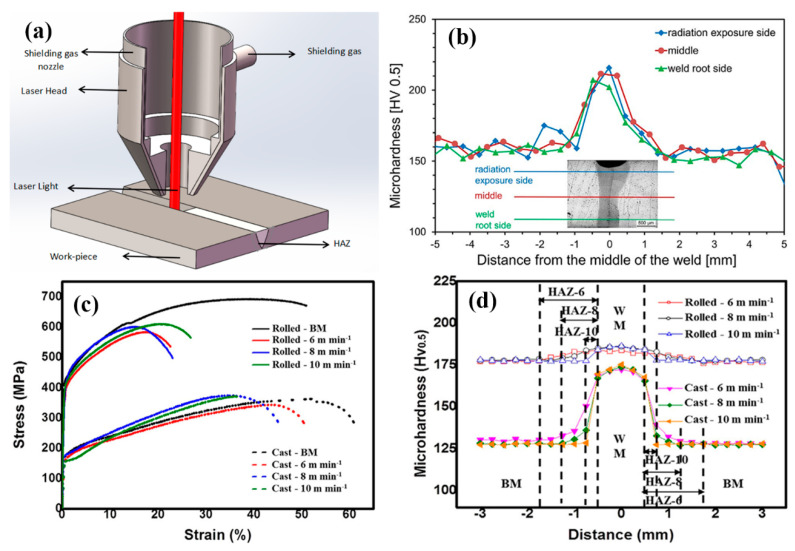
Hardness distribution in laser welded joints of HEAs: (**a**) Schematic diagram showing the laser welding process, (**b**) Microhardness profile of a butt–joint [26], (**c**) Stress-strain curves of the cast and rolled BM at various welding velocities [28] and (**d**) Hardness distribution in the transverse welds of the cast and rolled HEAs at various welding velocities: 6–10 m min^−1^ [28].

Research on laser beam welding (LBW) of HEAs has been reported in several recent publications [26,27,28,29,30]. In a laser welding process, pieces of metal are melted and joined through a concentrated heat source provided by the laser beam, forming a narrow, deep weld (Figure 4a). LBW has much higher welding rates than arc welding and is more suitable for high volume applications. LBM is based on keyhole or penetration mode welding [17]. Compared to arc welding, the heat density for laser is much higher and the welding point/zones is narrower and the cooling could be much faster [17,18]. Kashaev et al [26] studied LBW of a CoCrFeNiMn-type HEA produced by self-propagating high-temperature synthesis (SHS). The synthesis of the initial CoCrFeNiMn-type alloy was carried out with the use of thermite-type SHS powders containing oxides of the target elements (NiO, Cr_2_O_3_, Co_3_O_4_, Fe_2_O_3_, MnO_2_ and high purity Al as the metal reducer). The SHS-fabricated alloy was characterized by ∼2 times reduced Mn content in comparison with that of the other principal components and the presence of impurities including Al, C, S, and Si. In the experiments, welded butt-joints were produced using an 8.0kW fiber laser with a fiber optic (300 μm core diameter) and a 300 mm focal length with a welding speed in the range between 3.0 m/min and 6.0 m/min. Detailed metallurgical analysis revealed that the difference in microstructure and grain orientation distribution between the BM, HAZ and the FZ was not significant. As shown in Figure 4b, the FZ was not fully symmetric. Detailed Vickers hardness tests at the top, middle and bottom of cross-section showed a pronounced increase in microhardness from (153±3) HV 0.5 (BM) to (208±6) HV 0.5 (FZ) (Figure 4b). Nam et al [28] recently also reported the change of hardness on LBW of equiatomic Co0.2Cr0.2Fe0.2Mn0.2Ni0.2. The specimen in the work was prepared *via* vacuum induction melting, casting and homogenisation at 1100 °C for 24 h followed by air cooling. The tensile test data (Figure 4c) showed that the rolled specimen was much stronger with significant work hardening than the cast specimen. Systematic tests also showed that the stress-strain curves were not very sensitive to the change of welding speed, further details can be found in the paper [28]. Tests on the cross section of the weld of rolled specimen and cast specimen revealed hardness increase in the FZ and HAZ over the BM, but to a distinctively different extent. The hardness increase in the FZ of the rolled HEA specimen was limited while the hardness increase in the FZ of the cast specimen was over 30% (~130 to ~170 HV 0.5) (Figure 4d). Similar levels of hardness increase were also reported in the work on welding of CrMnFeCoNi plates with thickness of 1 or 2 mm [29], in which metallurgical analysis showed the FZ formed a dendritic structure with the dendritic and interdendritic regions relatively rich in Fe and Mn, respectively. The hardness of the FZ was much higher than the BM (~185 HV_0.1_ *vs.* ~143 HV_0.1_). Despite the difference in material systems and the condition of the BM, the thickness and welding powers and speeds [26,28,29], all these works showed an increase in the hardnesses of the FZ and HAZ.

**Figure 5:**
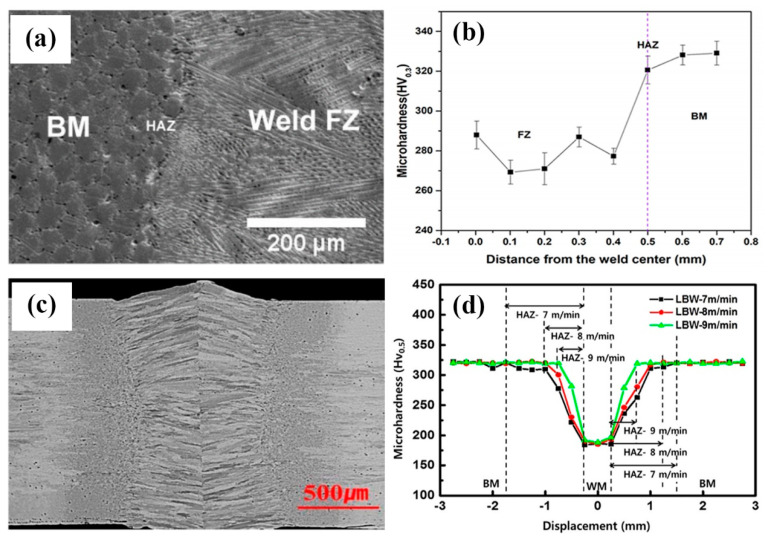
Structure and hardness of laser beam welded Al0.5CoCrFeNi HEAs: (**a**) Welding zones and boundary [30], (**b**) Microhardness profile on the surface [30], (**c**) Structure of the welded joints [27] and (**d**) Hardness distributions in the transverse weld for various welding velocities [27].

The trend of the other reported works on LBW showed a difference in terms of the hardness changes in the FZ [19,27]. In the work by Sokkalingam et al [19], 1 mm thick sheets of Al0.5CoCrFeNi were welded with a power of 1.5 kW and a traverse speed of 600 mm/min. The FZ that was exposed to rapid heating and cooling rate showed clear grain refinement (Figure 5a) with two kinds of dendritic structures: few longer columnar dendrites at the region next to fusion line, at the interloop boundaries and weld center with an average dendritic spacing of 4.8 μm and smaller equiaxed dendrites at in-between areas. Different from the results shown in Figure 4, Vickers microhardness test showed that the FZ became softer than the BM (Figure 5b). Similar hardness drop in LBW has also been reported by Nam et al [27] on Co0.2Cr0.2Fe0.2Mn0.2Ni0.2 (Figure 5c,d). In the work, the HEA slab used in laser butt-welding was homogenized at 1100 °C for 24 h and hot-rolled to 3 mm, followed by air cooling, then cold-rolled to 1.5 mm at room temperature (25 °C). As shown in Figure 5d the hardness of the FZ is much lower than the BM. The data also clearly showed that the width of the HAZ was affected by the welding speed but the bound of hardness of the FZ was not sensitive to the welding speed.

### 2.3. Electron Beam Welding

**Figure 6:**
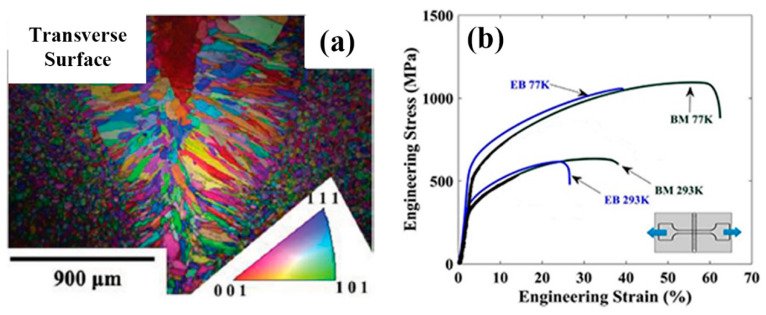
Structures and properties of electron beam butt–welded joints of CrMnFeCoNi alloy: (**a**) Electron backscattered diffraction (EBSD) maps showing the grain structure of the welded joints on transverse surface and (**b**) Tensile test results of welded joint [20].

**Figure 7:**
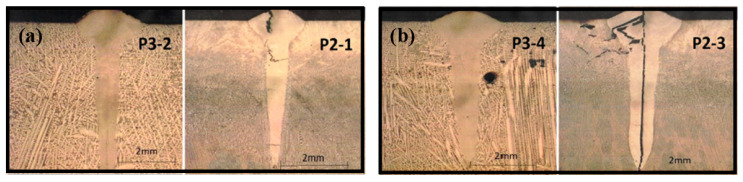
Structures of electron beam deep penetration welding of AlCrFeCoNi HEAs with different heat input: (**a**) 72 J/mm [32] and (**b**) 108 J/mm [32].

In an electron beam welding (EBW) process, a beam of high-velocity electrons is applied to two materials to be melted and joined. The heat density is high and the melted zone is normally thinner than that in laser and arc welding. In the work by Wu et al [31] on weldability of a high entropy CrMnFeCoNi alloy using a controlled test, a face centered cubic (FCC) CrMnFeCoNi alloy was selected. Welds produced by EBW showed no cracking. Tensile tests data showed that the welded joints possessed mechanical properties comparable to those of the BM at both room and cryogenic temperatures. Compared with the BM, deformation twinning was more pronounced in the FZ of the tested alloy. In another work, Wu et al [20] investigated low-heat-input EBW of CoCrFeMnNi HEA. The EB welds were made at a power level of 125 kV/2.2mA, and at a welding speed of 38 mm min^−1^. The work also showed that the welded specimen has maintained the strength and ductility of the BM indicating good weldability of the HEA in this condition (Figure 6). In the work by Nahmany et al [32], electron beam surface re-melting was employed to modify the surface properties of two five-component AlxCrFeCoNi HEAs (x-0.6 and 0.8) prepared by vacuum arc-melting. The effects of electron beam heat on the structure and mechanical properties of deep penetration welding of HEAs were investigated. The quality of weld was found to be dependent on the welding condition (Figure 7), when the welding heat input was increased from 72 J/mm (P2-1 and P3-2) to 108J/mm (P2-3, P3-4), cracks were observed, which is thought to be due to the residual stresses. In addition, the hardness of the FZ also increased relative to the BM, which is different from EBW. The reason for the hardness difference requires further studies but this highlighted the effect of depth and energy input on the quality of the welding process.

### 2.4. Friction Stir Welding

**Figure 8:**
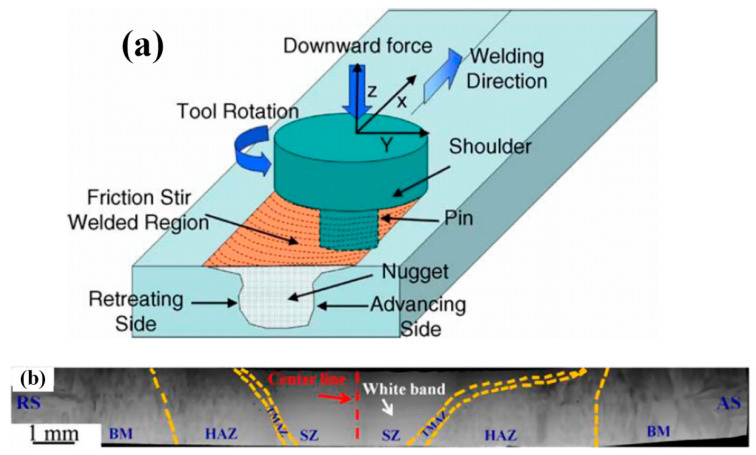
(**a**) Schematic diagram showing the friction stir welding (FSW) process and (**b**) Microstructure formed in FSW of Co16Fe28Ni28Cr28 alloys [34]: stir zone (SZ), thermomechanically affected zone (TMAZ), heat affected zone (HAZ) and BM.

Several works have studied the friction stir welding (FSW) of HEA systems [29,33,34,35,36,37]. Different from arc, laser and electron beam welding, FSW (Figure 8) is a form of solid-state joining, in which two facing workpieces are jointed through the heat generated by friction between the rotating of a non-consumable tool and the workpiece material [38]. The volume of the material affected in the welding process is much wider than other fusion based welding processes. Zhu et al [34] studied the FSW of a typical FCC CoCrFeNiAl0.3 HEA. The sample was made by arc-melting and cast into ingot plates with a dimension of 2 mm × 10 mm × 30 mm. The welding process was performed with speeds of 30 and 50 mm/min while the rotation rate and load force were kept at 400 rpm and 1500 kg, respectively. The tool has a shoulder diameter of 12 mm, probe diameter of 4 mm and probe length of 1.8 mm. The FSW joint consisted of four different regions: the stir zone (SZ), thermomechanically affected zone (TMAZ), HAZ and BM (Figure 8). The SZ exhibited refined grain size arising from recrystallization and it exhibited higher hardness due to grain size refinement. The TMAZ exhibited a mixed microstructure comprising coarse and fine grains due to partial recrystallization. The XRD results indicated that the HEA remained an FCC structure after FSW. The SZ showed a refined equiaxed microstructure due to recrystallization, and the hardness of the FZ was found to be much higher (220 HV) than the BM (180 HV). Another study [35] reported the work on FSW of a ductile Co16Fe28Ni28Cr28 HEA of a low content of Co with a particular focus on microstructural evolution and weld strength in comparison to typical FCC HEAs. The work revealed a similar trend of hardness increase in the SZ compared to the hardness of the BM (~250 HV vs. 150 HV). The work also showed that the grain size decreased slightly while the hardness increased with increasing the welding speed.

**Figure 9:**
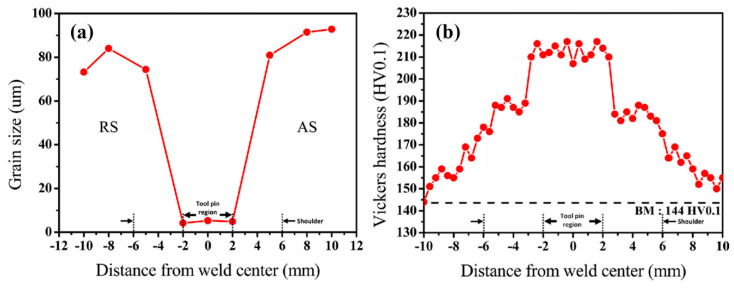
(**a**) Grain size at different distance from weld center positions and (**b**) Vickers hardness in the cross-section of FSW CrMnFeCoNi HEA [29].

Jo et al [29] investigated the microstructure and mechanical properties of friction stir welded CrMnFeCoNi HEA. The material was prepared by vacuum induction melting and hot rolling at 1100 °C. The dimension of the FSW plates was 55 mm × 60 mm × 2 mm. The pin diameter was 4–5.76 mm, the pin length was 1.85 mm, the shoulder diameter was 12 mm and the tilt angle was 3°. The FSW was carried out at a welding speed of 150 mm/min and tool rotation speeds of 600 and 70 RPM. Detailed metallurgical analysis showed that FSW refined the grain size in the weld region by a factor of ∼14 when compared with the BM (Figure 9a). The hardness in the weld region was much higher than the BM (~215 HV vs.144 HV) (Figure 9b). The tensile strength and ductility of FSW CrMnFeCoNi were comparable to that of annealed CrMnFeCoNi. This is probably associated with considerable microstructure refinement in the SZ and further recrystallization in FSW.

**Figure 10:**
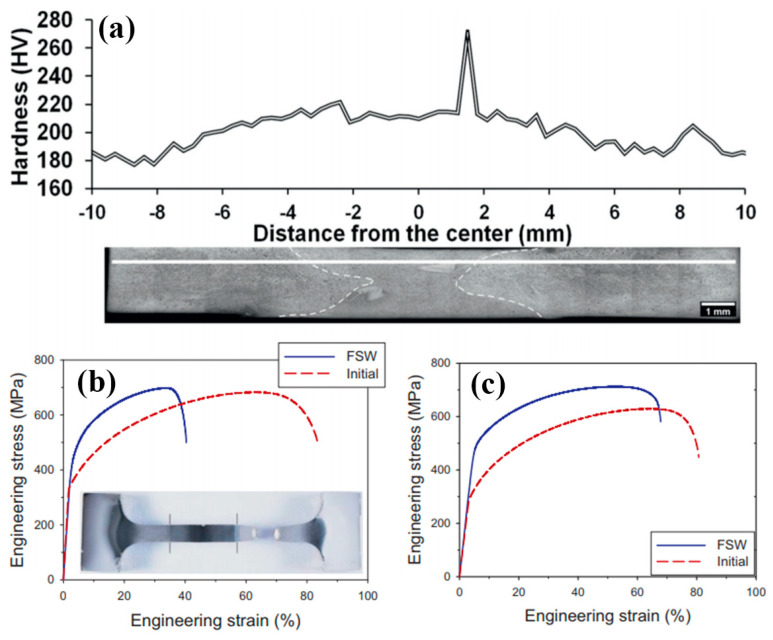
Microhardness distribution across the weld seam (**a**) and tensile stress-strain curves of the FSW specimens cut across (**b**) or along (**c**) the weld seam [36].

Shaysultanov et al [36] studied FSW of a carbon-doped CoCrFeNiMn HEA in butt joints. Along with the principal elements, a small amount (0.9 at.%) of C was added to the alloy produced by SHS. The CoCrFeNiMn alloy was produced using thermite-type SHS, in which a mixture of powders (oxides of the target elements NiO, Cr_2_O_3_, Co_3_O_4_, Fe_2_O_3_, MnO_2_, pure carbon C, and Al as the metal reducer) was used as the starting material. The as-cast alloy was cold rolled and annealed at 900 °C to produce a refined microstructure. The microhardness measurement showed an approximately 40 HV increase in the area of the SZ in comparison with the BM. Tensile tests were performed on samples perpendicular to the welded seam as well as on the sample along the welding direction (Figure 10). The data for both sample conditions showed a clear noticeable rise in strength in comparison with the BM. This can be associated with the microstructure refinement and some increase in the volume fraction of M_23_C_6_ carbides [36]. The sample with the loading axis perpendicular to the weld maintained around 50% of the ductility, while the sample fully taken from the seam showed comparable ductility to the BM.

## 3. Grain Structure, Element Distribution and Precipitations in Welded Structures of HEAs

The structures at different levels (grain, precipitation and lattice) are important to the integrity of the weldment. The structure, precipitation and element segregation/redistribution in a welding process are also important for some service conditions such as corrosion, creep etc. [1,12,15,16]. Apart from the difference in the general structure and hardness profiles of welding zones detailed in Section 2, research work on welding of HEAs also revealed significant difference in secondary phase and precipitation associated with different welding processes and material systems. In arc welding processes, such as GTAW, which is like a miniature casting process, the weld metal experiences a rapid cooling rate. The main grain structure is elongated columnar dendrites nucleated from the fusion line and equiaxed grains near the weld centerline [19,20]. Sokkalingham et al [19] has performed detailed XRD analysis on GTAW for Al0.5CoCrFeNi and found that both the volumetric fraction and lattice constant for both FCC and BCC phases change. The volume fraction of FCC increases (from 76 to 97.8%) but the lattice constant of FCC decreased from 0.3568 to 0.2424, and the lattice constant of BBC increased significantly, from 0.2132 to 0.3020. This is an interesting finding, and further research is required on how this is linked to the element segregation and how this change may affect the mechanical behaviour including shear and corrosion [39,40].

**Figure 11:**
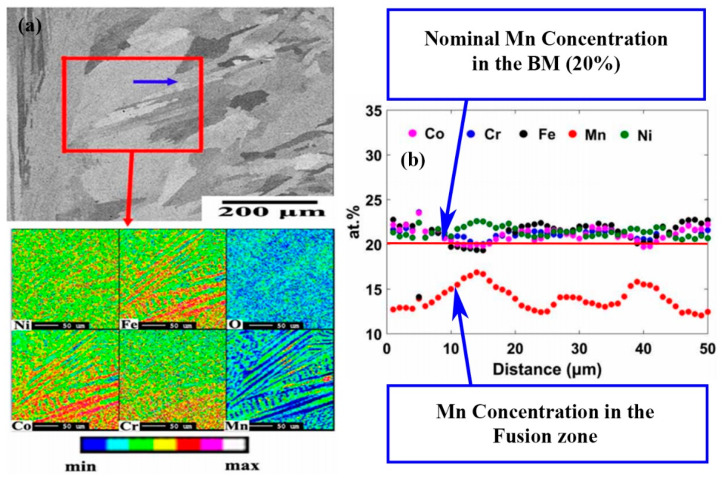
Elemental mapping of fusion zone (FZ) for electron beam welding of CoCrFeMnNi: (**a**) Microstructure of the electron beam (EB) weld zone area and electron microprobe analyzer (EMPA) compositional mapping of the marked area in upper figure and (**b**) Compositional profile along the arrow shown in (**a**) [20].

Composition distribution and element segregation across the welded joint at macro and micro scales are important to the understanding of the mechanical and corrosion resistance of the welded joint, which may be associated with many different mechanisms, such as evaporation, time and temperature for element diffusion, oxidation and precipitation, etc. [4,19,20,30,41,42,43]. For CoCrFeMnNi alloy [20], the GTA weld exhibited microsegregation behaviour in the Mn- and Ni-rich interdendritic region and Co-, Cr-, and Fe-rich dendrite cores, but the depletion of Mn was not observed, while for EBW weld, there was clear Mn depletion in the FZ (Figure 11) [20]. As shown in Figure 11b, the average value of the Mn is lower than the nominal value (20%). This was probably caused by evaporation of Mn due to the high power density associated with the EB welding process. For Al0.5CoCrFeNi, the elemental analysis in FZ revealed that the dentrites were rich in Fe and Co, depleted of Al and Ni, and had an even distribution of the Cr element [19]. Similar results have been observed in LBE of Al0.5CoCrFeNi [30]. It is not fully clear if evaporation of Al had played role influencing the structure and properties. However, elemental analysis showed that the concentration of Al element in dentrites and interdentrites of the as-welded (AW) sample is lower than the grains and the grain boundaries of the BM. The dendrites of the AW have a Al concentration of 4.78 %, while the grain of the BM has a concentration of 5.27%; The data also shows that the interdentrites of the AW region consist of 10.69% Al, while the Al concentration in the BM-grain boundary is about14.43%. So, it is possible that evaporation of Al may have occurred during the LBE process, which requires further quantitative studies together with other mechanisms proposed by the authors, such as precipitation of BBC phase (Al-Ni rich phase). In both material systems (i.e., AlCoCrFeNi and CoCrFeMnNi), the primary dendrite arm spacing and the extent of elemental segregation were less in the welds than in the cast ingot as the cooling in welding is much higher than in casting. In LBW, the heat density and the cooling rate is much higher than arc welding, the FZ gives the appearance of a columnar grained microstructure with random crystallographic orientation, and no significant change in microstructure near the FZ-BM boundary was observed [29]. In another work [26], the differences in microstructure and grain orientation distributions between the BM, HAZ and the FZ were not significant for CoCrFeNiMn-type HEA in LBW. For CrMnFeCoNi, the interdendritic and dendritic regions are enriched in Mn and Fe, respectively, similar to that at the FZ-BM boundary. LBW also resulted in precipitation of the nanoscale B_2_ phase particles for CoCrFeNiMn-type HEA. In the weldment of Al0.5CoCrFeNi, the equiaxed polygonal grains with grain size of 60 μm in homogenized state transformed to longer columnar dendrites at the region nearer to fusion boundary and inter-loop boundaries with an average dendritic spacing of 4.8 μm and fine equiaxed dendrites at the weld center on welding [19]. The work also found that, in the LB weldment, the hardening factor (Al-Ni rich BCC phase) was less in the weld metal than that in the BM, therefore the weld metal exhibited a lower hardness than that of BM.

**Figure 12:**
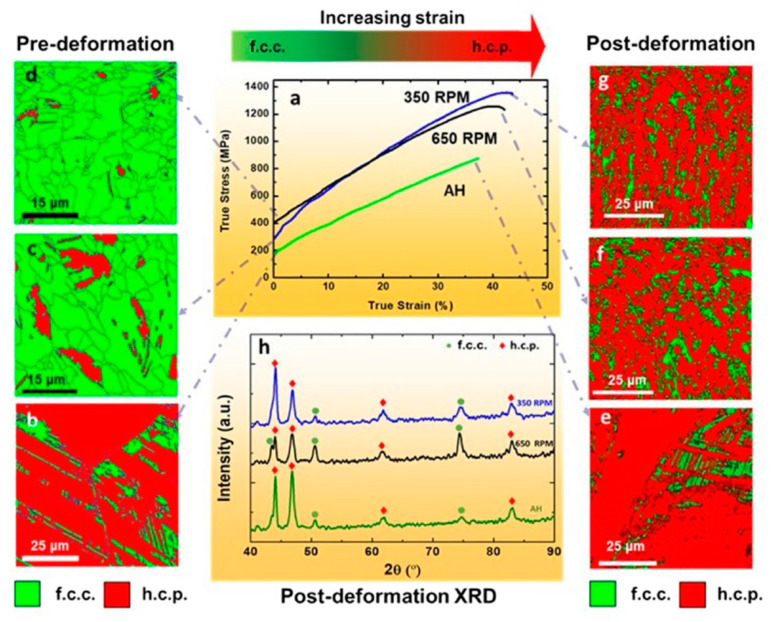
Structure and stress strain curves of high entropy alloys (HEAs) with enhanced properties through friction stir welding [45].

For FSW, the SZ comprised of refined grains arising from dynamic recrystallization. The HAZ exhibited a columnar structure [34,35]. In another work [29], EDS-line scan showed that in general the dendrites were enriched in Fe and depleted in Ni and Mn. The fluctuation in composition was significant: between ~5 and 15 at%. The cause for this compositional fluctuation in the FZ, which is expected to stay hot longer than the FZ-BM boundary and thereby allow for greater homogenization, is not clear and needs further study. In the work, relatively dark spherical particles were also detected in the High-angle annular dark-field image (HAADF) and all the element maps were found to be rich in Mn, S and O indicating that these were Mn sulfides/oxides. In the work on FSW of carbon-doped CoCrFeNiMn HEA [36], the microstructure of the SZ was shown to contain M_23_C_6_ carbides. More importantly, the increase in the volume fraction and size of the carbides (to 7% and 150 ± 62 nm, respectively) after FSW in comparison with that in the BM can be clearly noted. All these may have contributed to the strength and hardness increase of the welded zone in FSW as illustrated in Figure 9 and Figure 10. This suggests that, for FSW, there is no specific post welding processing needed to obtain a hardened joint, i.e., the alloy hardens due to the “natural” heat treatment during welding [36]. This is an advantage of interstitial alloying of HEAs. This indicates that higher strength of the FSW joints can be achieved in similar alloys by tailoring microstructure *via* adjusting the chemical composition and processing parameters even [26,33,44,45]. A typical example is shown in Figure 12, which shows that the FSW can be used for processing HEAs with enhanced strength and ductility in a friction stir processing engineered dual phase HEA [33].

## 4. Discussion and Future Works

The recent work summarized in Section 2 and Section 3 showed that HEAs have good welding characteristics. A broad range of welding methods were found to be applicable to the welding of HEAs and most cases showed no significant defects or cracking. The size of different welding zones varies with the welding method, such as electron beam, laser and arc welding. FSW resulted in a much wider FZ due to the involvement of the rotating pin. In most of the cases, the shape of the welded beam is symmetrical, which represents good weldability for structure and quality control. However, there are reported cases in which some butt-joints showed a non-symmetrical weld shape (Figure 4b), this is most likely due to coarse columnar grains of the base material [26]. Most of the reported works were based on butt-welding, which is one of the simplest forms of welding configuration. More complex setup such as tensile, shear, bending, torsion, and impact, in which the stress condition is more complex and close to real service conditions, need to be considered.The residual stresses also need to be studied systematically. Residual stress is influenced by many factors such as localized heating and cooling, differential volumetric change occurs both at macroscopic and microscopic level [46]. Limited work has been reported on the measurement of residual stresses in the work reviewed, which were mostly based on relatively simple, less constrained samples. In the case of EBM deep penetration welding of AlxCrFeCoNi HEAs, it was shown that residual stresses could cause cracking (Figure 7b). HEAs have greater freedom in designing composition, mechanical/physical properties, such as yield strength, stiffness, thermal conductivity and expansion, all these may offer opportunity to optimize the material design to manage the residual stresses in welding processes.

As detailed in Section 2 and Section 3, the works published clearly show that the change of the strength/hardness of the FZ and HAZ varies between the composition systems and the welding process employed. This is a significant difference from other engineering materials such as carbon or stainless steels, for which, the FZs normally have a hardness increase [47,48]. For nonferrous metals such as magnesium, the hardness for the FZ normally becomes higher but the hardness of the HAZ may drop [49]. For HEAs, hardness drop in the FZ was observed for Al0.5CoCrFeNi in laser and arc welding, while the hardness drop for CoCrFeMnNi is relatively limited or slightly increased. Sokkalingam et al [19,30] suggested that the hardness drop for the Al0.5CoCrFeNi in the LB weldment was due to the reduction of the hardening factor (Al-Ni rich BCC phase) in the weld metal than in the base metal. The processing method of the sample also influences the hardness changes. For example, as shown in Figure 4, the hardness change for the cast specimen after welding was much more profound than that for the rolled specimen. Other parameters such as welding speed and power also showed some influence on the hardness change but not as significant as the material groups and welding methods. Another issue, which requires further work, lies in the question how the hardness/strength change of the FZ and HAZ may affect the overall ductility and toughness of the welded joints. Hardness/strength drop is not an ideal situation in terms of strength following general engineering principles, however, this may potentially offer a way to enhance the ductility and toughness of the welded joints. This can be beneficial to some service condition where toughness is more critical than strength or hardness. Preliminary numerical modelling work by the authors (in-process, result not shown) indicates that soft FZs in HEAs may help to spread the load more evenly and reduce stress concentration, which commonly exists in welded joints with a hardened FZ [17], thus improve the overall ductility or toughness of the welded structure. In the work by Wu et al [31] on EBW of CrMnFeCoNi, tensile test results showed that, compared with the BM, deformation twinning was more pronounced in the FZ of the tested alloy. Further combined experimental and numerical modelling work is required to systematically investigate the structure and stress in the FZ, HAZ and the HAZ-BM or HAZ-FZ interface within welded joints of HEAs under controlled material, welding and testing conditions.

For HEAs and welded structures, many factors may contribute to the strength of the materials (such as grain refinement, precipitation, residual stress, volume ratio of different phases) [15,16,22,23,24,25,41,42,43]. Grain size is still an important but not necessarily the dominating factor depending on the HEA system and the welding processes utilized. For FSW, the SZ becomes much harder [19,20,26] than the BM. One major mechanism contributing to the hardness increase is the refinement of the grain, but the trend of the grain size effect does not fully follow the theories or data established for conventional bulk HEAs. For bulk HEA alloys, the Hall–Petch (H–P) trend between grain size and strength is applicable for a range of HEAs based on either tensile tests or hardness data including CoCrFeNi [50,51,52,53], CoCrFeNiMn [7,54,55,56] and AlxCoCrFeNi [57,58,59,60,61]. While for welding, the suitability of H–P relationship varies with the materials and welding conditions. For example, the data for FSW of Co16Cr28Fe28Ni28 [35] and Al0.1CoCrFeNi [34,62] followed the H–P trend well, but for arc welding and laser welding, the hardness and grain size relationship is opposite to the H–P trend [19,30]. These differences reflect that the controlling operative strengthening mechanisms are more complicated than pure grain refinement, and other factors such as phase change, precipitation all may affect the hardness to different extents. A more quantitative data driven methodology is required to clarify or establish the dominating mechanism(s). For example, the combined contribution of grain refinement and precipitation was quantified in [36] through combined Hall-Petch coefficient analysis. The work showed that a decrease in grain size by a factor of two (from 9.2 μm in the initial condition to 4.6 μm after FSW) resulted in an increase in strength by ~55 MPa. The rest of the increase in the yield strength (~145 MPa) was attributed to the precipitation strengthening associated with some increase in the volume fraction of the M_23_C_6_ carbides. Such quantitative analysis is not only important for establishing the strengthening mechanism, it is also essential to provide guidelines for developing effective pre- or post-treatments of welded structures. The published research work has successfully highlighted the key issues influencing the mechanical properties of the welded joints, which is important for expanding the applications of HEAs and welding processes. However, the data available is still limited to draw direct comprehensive conclusion on the property differences between different welding methods. Even though the general heat input is different between various welding techniques, but more systematic data on the cooling curves of comparable materials systemsare required to be conclusive when quantifying the main strengthening mechanisms. Physical simulation with controlled cooling (such as Gleeble^TM^) will help to quantify the effect cooling history on the microstructure and properties of different welding zones [62]. Establishment of the mechanisms of phase change, grain refinement and precipitation is also directly beneficial to the development of new materials and interruptive control technologies to maintain or improve the beneficial properties of HEAs such as high temperature stability and corrosion resistance of welded structures of HEAs [63,64,65].

## 5. Summary

Recent works on welding of HEAs with various welding methods of different setup and heat input were reviewed in detail focusing on the research on main HEA systems when applying different welding techniques. The structures and properties of the welding zones in particular the FZ and the HAZ formed with different welding methods were compared and presented in details and the structure properties relationships were discussed. The works showed that weldability of HEAs varies with the composition groups and the welding methods employed. Arc and laser welding of AlCoCrFeNi HEAs resulted in lower hardness in the FZ and HAZ and reduced strength. FSW resulted in higher hardness in the FZ and maintained the strength of the welded joints under tensile load. The welded of HEAs are capable to maintaining reasonable proportion of strength and the ductility. The key structural changes including element distribution, the volume fraction of FCC and BCC as well as some reported lattice were summarized and analyzed. The effects of evaporation for high energy welding (such as LBM and EBM) on composition and structure requires further quantitative study. Detailed mechanism(s) governing the mechanical properties including the contribution of the grain size-properties/hardness relationship in the form of Hall–Petch (H–P) effect for both bulk and welded structure of HEAs were discussed. Future research is required to establish the strengthening mechanisms of the welded joints and the effect of the observed hardness changes in the FZ on the strength and toughness of welded structures of HEAs. Such quantitative analysis will provide guidelines for developing effective pre- or post-treatments of welded structures. Studies of residual stress for different welding processes and pre-post weld treatments, in particular for under complex loading conditions, is required for enhancing the applications of HEAs. It is also beneficial to the development of new materials and interruptive control technologies to maintain or improve the beneficial properties of HEAs such as high temperature stability and corrosion resistance of welded structures of HEAs.
